# Obituary

**Published:** 1884-10

**Authors:** 


Obituary.—Died, in Columbus, Ohio, on Friday, August 22, Dr. Hiram Todd, aged 76 years. Dr. Todd was one of the oldest practitioners of dentistry in the State, and was, at one time, one of the most prominent.

Hiram Todd was born in Plymouth, Litchfield county, Conn., August 5, 1808. At an early age he gave evidence of being of an ingenious turn of mind, and when but a young man he went to New York city and engaged with a watch and clock maker. At the same time, he took up the study of dentistry for himself, and in 1838 opened an office in the great metropolis. The following year his old employer had occasion to come west and put up a clock in Detroit and one for the old Trinity Church, on Broad street, in this city. Knowing the skill of Mr. Todd, he asked him to accompany him on this trip and help place the clocks in position. After the work had been done the two started back for New York, but the trip was a long one in those days, and at Reynoldsburg, N. Y., Dr. Todd stopped, and, as he had brought his dental outfit with him, he opened an office there, but remained only a short time, when he retraced his way back to Columbus and resolved to make this his home. His success was great, and the Doctor has often remarked that his practice grew rapidly enough to satisfy the most ambitious of men.

On January 1, 1851, Dr. Todd was married to Miss Carrie E. Wright, of this city, who still survives him. The result of this union was three children, all of whom are living. They are Dr. Will H. Todd, a prominent member of his father's profession, Mrs. Will Stimson, and Miss Florence Todd.

For the past seven years Dr. Todd had suffered with paralysis, and for the past six weeks had been confined closely to his room. His death was not unexpected, as his advanced age rendered his constitution quite feeble.

